# Management Approaches for High-Risk Cutaneous Squamous Cell Carcinoma with Perineural Invasion: An Updated Review

**DOI:** 10.1007/s11864-024-01234-z

**Published:** 2024-08-05

**Authors:** Martina Catalano, Filippo Nozzoli, Francesco De Logu, Romina Nassini, Giandomenico Roviello

**Affiliations:** 1 Section of Clinical Pharmacology & Oncology, Department of Health Sciences, University of Florence, Florence, Italy; 2 Section of Anatomic Pathology, Department of Health Sciences, University of Florence, Florence, Italy

**Keywords:** Cutaneous squamous cell carcinoma, Perineural invasion, Adjuvant therapy, Surgery

## Abstract

Cutaneous squamous cell carcinoma (cSCC) stands as the second most prevalent non-melanoma skin cancer worldwide, comprising approximately 20% of all cutaneous malignancies. Determining its precise incidence poses challenges; however, reports indicate a global increase in its prevalence. At the time of diagnosis, the majority of cSCCs are localized, resulting in favorable 5-year cure rates surpassing 90%. Nevertheless, a subset of patients (3–7%) encounters locally advanced or metastatic cSCC, leading to substantial morbidity and mortality. The risk of metastasis ranges from 0.1% to 9.9%, carrying an associated mortality risk of 2.8%. Factors influencing recurrence, metastasis, and disease-specific mortality underscore the significance of perineural invasion (PNI) as a key indicator. Patients with PNI may manifest clinical symptoms and/or radiologic signs of PNI, while the majority remain asymptomatic, and PNI is frequently identified upon histologic examination. Despite its lower frequency compared to other cancer types, PNI serves as a recognized adverse prognostic factor for cSCC. Surgery is the elective treatment for these patients, while the role of adjuvant radiotherapy (ART) is yet contentious and have not been conclusively assessed, particularly in clear surgical margin. Prospective comparative studies are required to comprehensively evaluate the benefit and the risks of ART for cSCC and PNI patients.

## Introduction

Cutaneous squamous cell carcinoma (cSCC) is the second most common non-melanoma skin cancer, constituting about 20% of global cutaneous malignancies [1, 2]. Determining its frequency precisely poses challenges, but reports to the World Health Organization indicate a worldwide increase in occurrence [3], with an annual rate of about 100 cases per 100,000 individuals in the United States [3, 4].

cSCC originates from epidermal keratinocytes, primarily affecting individuals with fair or light skin. Contributing factors include fair complexion, UV radiation exposure, advanced age, male gender, immunosuppression, smoking, and genetic predisposition [5, 6]. Most cSCCs are diagnosed in a localized state, with over 90% cure rates within 5 years [7]. However, a subset of patients faces locally advanced or metastatic cSCC, with 3–7% reporting metastasis, leading to significant morbidity and mortality. High-risk variations can exhibit metastatic rates as high as 37% [8].

High-risk cSCC, staged as N0 (lacking detectable regional lymph nodes) and M0 (devoid of distant metastasis), presents features linked to an elevated risk of local recurrence and metastasis [9]. For instance, high-risk cutaneous SCC in the head and neck region has a risk exceeding 5% for recurrence, lymph node metastasis, and distant metastasis due to patient and primary lesion characteristics. A significant proportion of cSCC metastases occur within the initial two years, predominantly involving locally invasive cSCC or nodal metastases [10].

Among factors linked to recurrence, metastasis, and mortality, perineural invasion (PNI) is highlighted as an indicator of heightened malignant potential [11]. PNI involves the microscopic identification of tumor proximity to nerve fibers, with the underlying mechanism remaining largely undisclosed. The incidence of PNI varies across cancer types, being infrequent in skin carcinoma (approximately 2% of basal cell carcinomas and 3% of HNcSCC). Individuals affected by PNI report sensory disturbances, emphasizing the need for early detection and improved risk assessment to reduce morbidity and mortality.

## Perineural Invasion in cSCC

PNI is defined as the invasion of tumor cells into the perineural space of a peripheral nerve. This process is commonly held responsible to precede the perineural spread (PNS) that is by definition more advanced, being radiologically or clinically apparent, which takes place when tumor cells spread along the peripheral nerve within the perineural space away from the initial point of invasion [12]. PNI can be considered into two categories: clinical PNI (cPNI) and incidental PNI (iPNI). cPNI is qualified as evidence of spread along large caliber nerves, radiologically detected, with magnetic resonance imaging (MRI) recognized as the most sensitive imaging technique for diagnosis [13], or clinically significant. The definition of cPNI is particularly relevant in the head and neck region where critical cranial nerves such as the facial and trigeminal nerves can be involved, resulting in symptoms such as pain, paresthesia, anesthesia, or paralysis. iPNI is defined as invasion of nerves identified by histological examination and represents small caliber nerves involvement. It is estimated that between 60 and 70% of patients with PNI present with incidental findings [14]. However, there is no unanimous agreement on the histopathological definition of PNI and its assessment remains controversial [15–18].

Karia et al. conducted a comprehensive meta-analysis, encompassing a total of 640 tumors in 622 patients, which examined cases exhibiting either cPNI or iPNI [19]. Predominantly, the cSCC tumors (67%) were localized in the head and neck region. Among the cases examined, 20.8% of the cSCC instances were recurrent at the time of data collection. The analysis revealed no statistically significant variance in the risk of nodal and distant metastasis based on the PNI classification. However, it was observed that patients with cPNI faced a notably elevated risk of local recurrence and death attributed to cSCC in comparison to those with iPNI. Specifically, the figures indicated that patients with cPNI experienced higher rates of local recurrence (37% *vs.* 17%; p < 0.001) and mortality due to cSCC (27% *vs*. 6%; p < 0.001) than their iPNI counterparts. Conversely, individuals with iPNI exhibited a superior 5-year disease-specific survival (DSS) rate when compared to those with cPNI (DSS, 88% *vs.* 70%; p = 0.002). Moreover, the overall 5-year OS rate was found to be higher for patients with cPNI than those with iPNI (66% *vs*. 43%; p = 0.003). It should be noted that the study cohorts comprised predominantly of elderly patients, who are inherently at an augmented risk of mortality due to numerous factors beyond cSCC.

Conversely, Cohen et al. recently presented retrospective data based on 104 patients diagnosed with HNcSCC, of which 61 exhibited PNI [20]. Histopathological examination revealed that 28 lesions displayed complete encirclement of nerves, 10 involved more than five nerves, and 12 implicated named nerves. Notably, patients presenting with facial weakness and positive surgical margins exhibited an elevated probability of histopathologic PNI. The study demonstrated a poorer disease-free survival (DFS) outcome in patients with PNI (p = 0.004), advanced tumor staging (p = 0.049), positive margins (p = 0.014), and involvement of more than five nerves (p = 0.0061). Histopathologic PNI, particularly when more than five nerves were involved, emerged as a predictor of DFS (hazard ratio [HR], 3.07; 95% CI, 0.33–1.38; p = 0.0061) both in the overall cohort and among patients with cPNI (HR, 3.43; 95% CI, 1.65–7.10; p = 0.00091). These findings suggest that cPNI may serve as a more potent prognostic indicator for survival than PNI in its entirety. Furthermore, DFS was markedly worse in patients displaying PNI, facial nerve weakness, advanced T stage, positive margins, and multiple nerve involvement.

These findings underscore the critical importance of meticulously assessing signs and symptoms of PNI during the initial clinical evaluation. Patients with cPNI may necessitate more aggressive treatment modalities and vigilant monitoring for potential recurrence post-treatment. It is advisable to consider continued long-term surveillance, employing MRI every six months for a duration of two to three years following treatment (refs if available). The decision should be tailored to individual patient preferences, age, and concurrent medical conditions. Timely detection and subsequent intervention hold the potential to enhance overall survival, although the precise efficacy of radiological surveillance remains insufficiently quantified.

## Staging Systems and Risk’ Categories

The diagnosis of cSCC relies on clinical characteristics, confirmed through histological analysis. In cases with suspicious lesions, it is crucial to perform a biopsy or excision for histopathological confirmation [7]. This procedure is essential for accurate prognostic classification and appropriate therapeutic management.

Existing standards for cSCC classification are embodied in the AJCC and BWH systems [10, 21]. According to AJCC 8th edition staging, tumors are categorized based on size, with T3 upstaging indicating high-risk features [22]. Confirmed high-risk histologic features include PNI, tumor thickness > 2 mm, Clark level IV or V invasion, primary site on the ear or lip, and poor differentiation [23]. UICC classifies tumors primarily based on diameter, while BWH considers risk factors to determine T classification.

European guidelines propose intrinsic high-risk factors, including clinical diameter, localization, thickness, poor differentiation, desmoplasia, PNI, bone erosion, and immunosuppression. Positive surgical margins are considered an extrinsic high-risk factor [7].

NCCN guidelines distinguish between low and high-risk cSCC, with high-risk attributes encompassing advanced local disease, regional lymph node involvement, PNI, recurrence, and immunosuppression [24]. NCCN further differentiates between "high-risk" and "very high-risk" based on distinct pathological features, such as the presence of tumor cells within the nerve sheath of a nerve located deeper than the dermis or measuring ≥ 0.1 mm [24].

The variability in high-risk factors emphasizes the need for further empirical evidence to establish their correlation with prognosis. Comparison between different staging systems and their risk based on PNI is summarized in Table 1.

**Table 1:** Summary of tumor risk classification based on the perineural invasion

Staging system (year), ref	Perineural invasion definition	Risk class
NCCN (Version 1.2024), [24]	Perineural invasion-dermal only (nerve diameter < 0.1 mm) Tumor cells within the nerve sheath of a nerve lying deeper than the dermis or measuring ≥ 0.1 mm (large PNI)	High-risk Very high-risk
AJCC 8th Edition (2017), [22]	Tumor cells in the nerve sheath of a nerve lying deeper than the dermis or measuring ≥ 0.1 mm in caliber or presenting with clinical or radiographic of named nerves	High-risk
BWH (2019), [21]	Perineural invasion of nerve(s) ≥ 0.1 mm in caliber or tumor invasion beyond subcutaneous fat	T2a-T3*
BAD (2020), [25]	Perineural invasion-dermal only (nerve diameter < 0.1 mm) Perineural invasion present in named nerve; nerve diameter ≥ 0.1 mm; or nerve beyond dermis	High risk Very high risk

*NCCN* national comprehensive cancer network, *AJCC* American joint committee on cancer, *BWH* Brigham and women's hospital, *BAD* British association of dermatologists. *based on the number of risk factors

## Management of cSCC with PNI

Current prognostic indicators for recurrence, pivotal in determining the appropriate course of definitive or adjuvant therapies, are grounded in clinicopathologic attributes expounded within solitary-center or expansive clinical investigations, or consensus conventions. These indicators have been formulated by assimilating local staging, site, depth, and pathological attributes.

### Surgical Treatment

Surgery holds its place as the foremost therapeutic intervention in addressing cSCC. Main treatment objectives revolve around complete elimination of the tumor while preserving functional integrity and favorable cosmetic outcomes. In scenarios where surgical intervention is unfeasible, such as cases involving locally advanced disease or elderly patients with concurrent medical conditions, radiotherapy (RT) is the primary therapeutic strategy [26]. Prevailing clinical guidelines provide clear and definitive recommendations regarding primary treatments for high-risk cSCC. The objective of surgical excision is to achieve both clinical and microscopic comprehensive resection (R0 surgery), securing unambiguous (negative) histological margins [27].

In accordance with the European guidelines, a clinical safety margin of 5 mm is advised for lesions categorized as low-risk [27]. On the other hand, cases of high-risk cSCC call for a clinical safety margin ranging between 6 to 10 mm, or alternatively, an approach involving micrographically controlled surgery (MCS). MCS entails surgical excision followed by horizontal section processing of skin tissue, subjected to microscopic examination. This iterative process continues until the absence of cancerous cells is ascertained at the surgical margins, based on anatomical documentation. Within the realm of MCS, methodologies like Mohs micrographic surgery (MMS) and 3D histology are embraced, utilizing frozen sections and paraffin sections respectively, to analyze tissue specimens [27]).

Chung et al. investigated the occurrence and risk factors associated with histopathologic upgrading of cSCC during MMS [28]. The term "upgrade" was defined as the identification of a less advanced degree of differentiation (poor or moderate) and/or the presence of bone or perineural invasion during MMS, which had not been initially detected in the histopathological evaluation of the initial biopsy. Among the 1558 tumors that underwent examination, 115 (7.4%) were subjected to upgrading during MMS. Through a comprehensive analysis involving multivariate logistic regression, it was found that male gender, prior field treatment, location on the ear/lip, rapid cSCC growth, and a tumor diameter exceeding ≥ 2 cm were all notable predictors of tumor upgrading. Those tumors that experienced upgrading appeared to necessitate more than three stages of MMS for adequate clearance, intricate closure methods such as flaps or grafts, or referral-based repairs.

Soleymani et al*.,* observed that the cohort of high-risk cSCC cases treated with MMS exhibited decreased incidences of local recurrence (LR), nodal metastasis (NM), and disease-specific death (DSD) in comparison to historical reference controls [29]. This assessment was conducted by utilizing both the staging systems of BWH and the AJCC 8th edition. The study also revealed that MMS confers a survival advantage specific to the disease over the historical approach of wide local excision in the management of high-risk tumors. Furthermore, the enhancement of local tumor control with MMS appears to contribute to a reduction in the occurrence of regional metastatic disease, and it is plausible that MMS might confer a survival benefit even for patients who develop regional metastases [29]. An extensive meta-analysis by Fraga et al. underscores a diminished rate of locoregional recurrence associated with complete margin assessment in comparison to sectional assessment [30]. The discrepancy is particularly pronounced in cases of high-risk keratinocyte carcinomas and cSCC characterized by PNI. Therefore, the strategy of peripheral and deep end face margin assessment (PDEMA), advocates for excision in combination with meticulous histological evaluation of the peripheral and deep excision surgical margins, positioning it as the standardized therapeutic protocol.

While varying guidelines put forth differing recommendations concerning safety margin dimensions for high-risk cSCC, a consensus exists that wider margins are imperative for high-risk cases as opposed to low-risk tumors. Recommendations regarding margin sizes are primarily rooted in expert consensus, retrospective studies, and observational analyses. The overarching goal remains the achievement of negative surgical margins whenever feasible, with the consideration of re-excision in cases of positive margins, as deemed practicable.

### Adjuvant Therapy

**Fig. 1:**
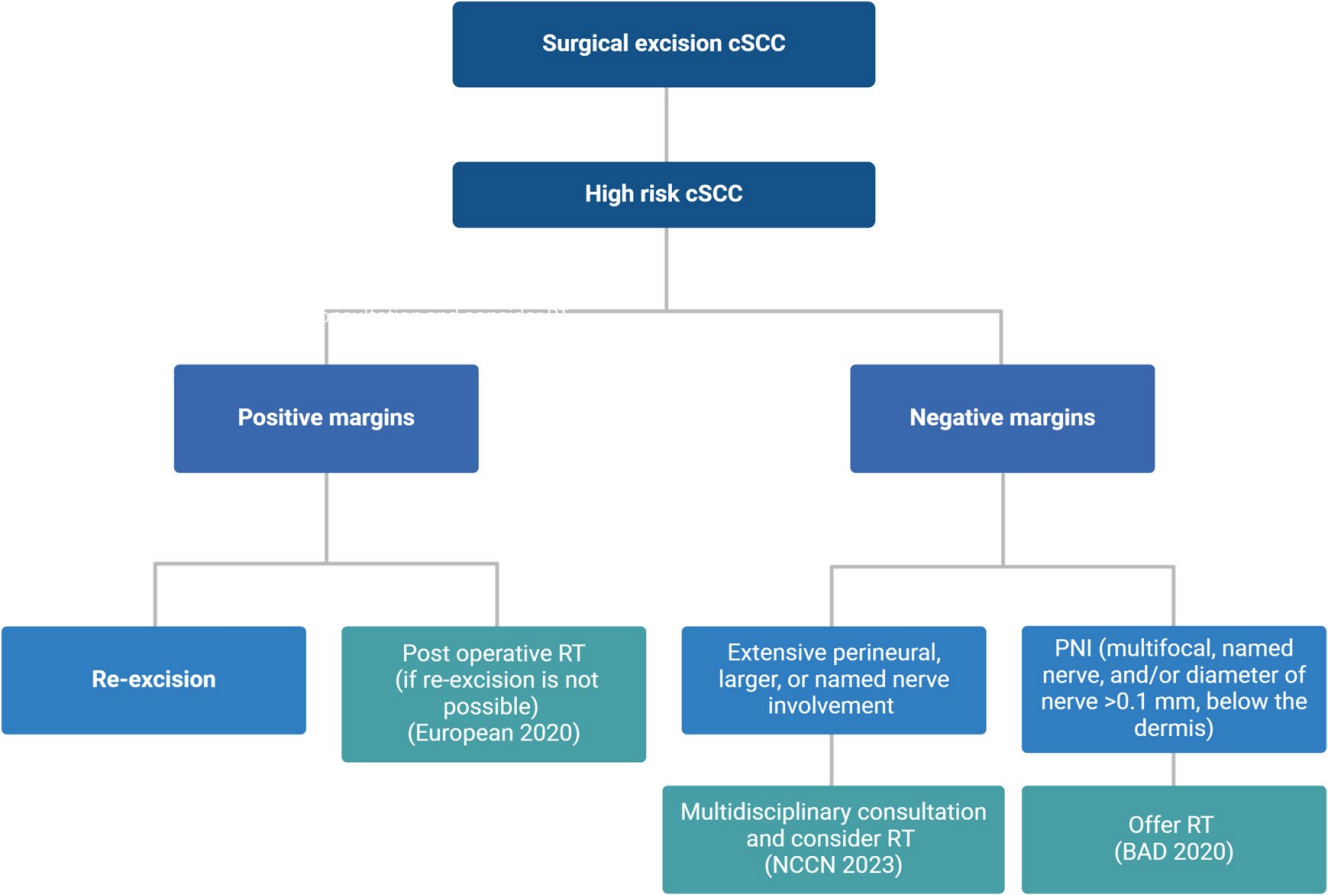
Current adjuvant radiotherapy recommendations for high-risk cutaneous squamous cell cancer. *Created with BioRender.com*

Adjuvant radiotherapy (ART) has been regarded as a treatment option for patients with high-risk cSCC (59). Figure 1 summarize the recommendations on ART in current guidelines. Nevertheless, the advantages of ART, particularly after achieving clear surgical margins, remain contentious and have not been conclusively assessed. Thus, substantial variation in the application of ART for cSCC. Certain studies reported in this analysis did not demonstrate a significant difference in survival outcomes between surgery and a combined approach involving surgery and ART (Table 2).

**Table 2:** Adjuvant radiotherapy in cSCC

Authors	Type of study	Population	N of patients	Intervention	Outcomes	Findings
Ebrahimi et al	Retrospective study	Patients with metastatic cSCC (a single parotid gland or neck nodal metastasis 3 cm)	168	Surgery ± ART	5 y DFS for patients treated with surgery alone: 97% RCR:87% vs. 91% with or without ART (p = 0.054)	No 5 y DSS difference between surgery alone group and surgery plus ART group
Zhang et al	Systematic review and meta-analysis	Patients with cSCC	2605	Surgery ± ART	ART reduce recurrence (OR, 0.56; p = 0.006) and prolong DFS (OR, 2.17; p = 0.008) and OS (OR: 2.94; p < 0.0001) in comparison with surgery alone ART resulted in a non-significant benefit in DSS (OR, 1.35; p = 0.76)	In cSCC patients with risk factors, including metastasis to the parotid gland, PNI and immunosuppression, the use of ART may be beneficial irrespective of surgical margin status
Kim et al	Meta-analyses	High-risk cSCC patients	3867	Surgery ± ART	LR for the surgery alone vs. the surgery plus ART: 15.2% vs. 8.8%; RM: 11.5% vs. 4%; DM: 2.6% vs. 1.7%; DSD: 8.2% vs.19.7%	There are no significant differences in poor outcomes between the surgery only group and the surgery with ART group
Stevenson et al	Retrospective study	Patients with SCC with PNI	31	Surgery ± ART	5-year DFS for patients treated with ART vs. those who did not receive ART: 100% vs. 68.8%	ART led to better DFS in cases of large-caliber PNI and small-caliber with other high-risk factors
Jambusaria-Pahlajani et al	Systematic review	High-risk SCC patients	2449 (74 with PNI)	Surgery ± ART	No significant difference in outcomes of PNI cSCC between those treated with surgery and surgery plus ART	In cases of PNI, the extent of nerve involvement appears to affect outcomes, with involvement of larger nerves imparting a worse prognosis
Garcia-Serra et al	Retrospective study	Patients with microscopic or clinical PNI of SCC	135	Radiotherapy ± surgery	5-year local control rates without salvage therapy in microscopic PNI vs. clinical PNI: 87% vs. 55%	Risk of local recurrence more than doubled in cases with advanced PNI (symptomatic or radiologically confirmed)
Canueto et al	Retrospective study	cSCCs with PNI	110	Surgery + ART	The risk of DSPOs is 0.328 times in primary cSCC with PNI managed with ART (p = 0.006) The risk of DSPOs is 0.248 times for patients managed with nerves involved ≥ 0.1 mm ART (p = 0.008)	Postoperative radiotherapy improved outcomes for cSCC with PNI (mainly if large caliber) and positive margins

Cutaneous squamous cell carcinoma, *cSCC* adjuvant radiotherapy, *ART* perineural invasion, *PNI* regional control rate, *RCR* local recurrence, *LR* regional metastases, *RM* distant metastases, *DM* disease-specific deaths, *DSD* disease-free survival, *DFS* disease-specific poor outcome events (DSPOs)(include local recurrence, metastasis (either to the parotid gland or to the lymph nodes), in-transit metastasis and disease-specific death)

In the study by Ebrahimi et al., the regional control rate stood at 91% *vs.* 87% (p = 0.054), and the average number of adverse features was 1.1 *vs.* 1.3 in the surgery-only and combined therapy groups, respectively [31]. However, this study revealed that 80% of patients with close margins, extracapsular spread (ECS), or other risk factors received ART, whereas over half of those treated solely with surgery did not have ECS. In other studies, ART was administered to patients with regional metastasis; this context potentially contributed to the absence of significant outcomes in these studies, given the potentially worse patient condition in the ART group [32, 33]. The pooled analysis conducted by Zhang et al., suggests potential benefits of ART in reducing recurrence rates and enhancing survival, both in the general population and in the subgroup with clear surgical margins [34]. Notably, studies demonstrating a survival benefit after ART often featured metastatic cSCC involving the parotid gland or regional lymph nodes, a common risk factor for significantly worse survival.

A meta-analysis by Kim et al. investigated the comparative risk of unfavorable outcomes among patients subjected to surgical intervention as opposed to those receiving both surgical treatment and ART [35]. The study encompassed a compilation of thirty-three investigations, enlisting a collective cohort of 3867 cases featuring high-risk cSCCs. They showed that for individuals harboring high-risk cSCCs who underwent margin-negative resections, the distinction in unfavorable outcomes between those subjected to surgical procedures and those receiving surgery with adjunctive radiotherapy was not different. However, retrospective analyses have underscored the efficacy of ART in cSCC patients with negative surgical margins, especially in cases featuring T3 stage, PNI > 0.1 mm, and poor histopathologic differentiation. Particularly, results hint at a potential reduction in the risk of local recurrence when combining surgery and ART (2.8%) compared to surgery alone (27.9%) in cases where PNI is the primary high-risk factor, although this difference did not reach statistical significance. To note that most studies that included cases with PNI focused on instances of large-caliber nerve invasion or cases with clinical and/or radiologic evidence of PNI. However, while a correlation between vascular involvement and positive surgical margins has been established as a risk factor for increased distant or regional recurrence, and a beneficial role of ART is suggested, data regarding survival outcomes linked to PNI nerve diameters or numbers are scarce and conflicting.

Stevenson et al*.* investigated individuals who had been diagnosed with histologically confirmed cSCC and evidence of PNI [36]. All patients were evaluated for eligibility to supplementary radiotherapy based on the identification of PNI characterized by large-caliber dimensions (≥ 0.1 mm in diameter), or PNI characterized by small-caliber dimensions (< 0.1 mm), coupled with other high-risk features such as dimensions exceeding 2 cm, limited differentiation, incursion surpassing subcutaneous adipose tissue, immunosuppression, and localization to the craniofacial region. Overall, 31 patients underwent MMS, achieving negative margins in all instances. Although the radiation oncologist recommended adjuvant radiotherapy for all patients, merely 15 individuals (48.4%) underwent the full course of treatment. The tolerability of ART was observed to be favorable, with 6 patients developing mild dermatitis. Over the span of a 5-year follow-up, no instances of local recurrence were recorded. A notable difference emerged between the two groups concerning nodal metastasis: all 5 patients who developed nodal metastases did not receive ART, whereas none among those treated with combined therapy exhibited nodal metastasis (p = 0.02). The 5-year period prognosis for DFS was different between individuals subjected (100% 95% CI, 100%-100%) or not subjected to ART (68.8%, 95% CI, 60.9%-76.7%). Large-diameter PNI was reported by 80% patients with nodal metastasis. However, one patient who underwent only surgical intervention and subsequently manifested nodal metastasis exhibited perineural invasion of smaller dimensions. These findings emphasize the plausible utility of ART for both cases involving large-caliber PNI and instances of small-caliber PNI associated with other high-risk attributes. While it was previously held that the caliber extent of involved nerve fibers could influence outcome, recent insights reveal that even instances of small-caliber PNI entail a fourfold increase in the likelihood of nodal metastasis if coupled with two additional risk factors The risk undergoes a further 14-fold increase if associates with three other risk factors [37].

Jambusaria-Pahlajani et al*.* compared the outcomes of high-risk SCC treated with surgical monotherapy to those subjected to a combination of surgery and ART [38]. Among the 2,449 instances of high-risk SCC considered, a subset of 91 cases was managed with surgery and ART. Within the 74 cases with PNI, outcomes encompassing local recurrence, distant metastasis, and disease-specific mortality were not different between the surgery and surgey plus ART. Analyzing the subset of 943 high-risk SCC cases featuring well-defined surgical margins, the risks associated with local recurrence, regional metastasis, distant metastasis, and disease-specific mortality were quantified at 5%, 5%, 1%, and 1%, respectively. Favorable therapeutic outcomes were reported for high-risk cutaneous SCC cases when clear surgical margins were achieved. Current available data do not offer specific high-risk features of patients who may benefit by ART. The extent of nerve involvement was found to impact on outcomes, as larger nerve involvement (diameters ≥ 0.1 mm) contributed to a worsened prognosis [38].

In another study focusing on PNI in cSCC conducted by Garcia-Serra and colleagues, it was determined that among 76 patients with advanced PNI, as defined by clinical symptoms or radiological findings, the risk of local recurrence was more than doubled (45%) when contrasted with 59 patients exhibiting only microscopic PNI (13%) [39]. Notably, this trend held true irrespective of the status of surgical margins. For cases featuring solely microscopic invasion, nearly all instances of recurrence were observed in cases with positive surgical margins. This underscores the significance of achieving clear surgical margins in yielding positive outcomes for microscopic PNI. However, this study does not provide a conclusive assessment of whether adjuvant radiotherapy further improves outcomes in cases with clear surgical margins, as all patients in this study received such treatment. Conversely, instances characterized by advanced PNI exhibited a substantial likelihood of recurrence even in the presence of clear surgical margins. This underscores the challenge of effectively managing advanced PNI solely through surgical intervention. Consequently, ART is generally recommended in cases featuring symptomatic or radiologically confirmed PNI.

In their most recent work, Canueto et al. conducted a comprehensive assessment of ART in cSCC with PNI. The primary objective was to identify the specific patient signatures that may better benefit of a specific therapeutic approach [40]. The retrospective analysis of a multicenter cohort comprising 110 instances of cSCC with PNI investigated the categories of PNI associated with unfavorable outcomes, evaluated the efficacy of ART in distinct subsets of cSCC with PNI, and examined the utility of ART with respect of the status of surgical margins (clear or positive). The outcomes showed that ART was superior to a strategy of observation alone, particularly for cases of cSCC with PNI and positive margins post-surgery. In patients undergoing observation alone the risk of adverse outcome events increased by a factor of 2.43 times (p = 0.025). This effect was particularly evident in cases withy positive margins and PNI measuring ≥ 0.1 mm (the likelihood of a negative prognosis increased eightfold, p = 0.0065). Notably, the implementation of ART yielded significant enhancements in long-term outcomes for patients afflicted with cSCC featuring PNI and positive margins following surgical intervention. However, the benefit of ART was less pronounced for cases with clear margins, particularly those with PNI in small-caliber nerves.

Although ART can lead to improved survival outcomes, it is also associated with increased post-treatment complications, such as skin erythema, mucositis, recurrent cellulitis, chronic pain, and others, severely impacting the patients' quality of life, or contributing to disease-specific death. Further prospective comparative studies are required to comprehensively evaluate the benefit and the risks of ART for cSCC patients.

## Future Directions

In recent years, PNI studies have focused on identifying new biomarkers related to the pathogenetic mechanisms of PNI. The goal is to contribute to more precise prognostic stratification and potential targets for future therapies. Existing stratification tools have had limited impact, particularly among high-risk cSCC patients. It is crucial to detect PNI-related biomarkers with both prognostic and predictive significance.

PNI is currently understood as occurring through invasion, involving a reciprocal association process between tumor cells and nerve components [26]. Various neurotrophic agents, including nerve growth factors and adhesion molecules, play a role in this process [41].

Wysong et al. developed a prognostic 40-gene expression profile (GEP) test for high-risk cSCC, revealing positive predictive value [42]. Eviston et al. evaluated the GEP of HNcSCC cases with PNI, showing significantly different profiles in those likely to develop recurrence [43]. However, the retrospective nature of the study limits its predictive value.

Warren et al. focused on expression analysis of PNI specimens, emphasizing mutations affecting p53 activation [44]. The analysis showed p53 activation signatures in tumors with PNI compared to those without.

In 2020, a focus on the expression of Melanoma Antigen Family A, 3 (MAGE-A3) at the mRNA level in cSCC with PNI was proposed [45]. Upregulated expression of MAGE-A3 emerged in poorly differentiated cSCC with PNI, suggesting a role in cancer progression. Larger studies are needed to validate its prognostic significance.

Zilberg et al. studied somatic mutations associated with adverse histopathological features in high-risk HNcSCC, finding exclusive FGFR2 mutations in patients with PNI [46]. These mutations are targetable, providing potential treatment options.

Despite efforts to understand biomarkers associated with PNI, data on large case studies are still lacking, especially regarding prognostic and potentially predictive value.

## Conclusion

This review aims to provide an updated insight into high-risk cSCC with PNI outlining the current, albeit undefined, management approaches for advanced cSCC with PNI.

The implementation of clinical/radiological evaluation to ameliorate the diagnosis of patients with PNI may add benefit to personalized treatment selection and follow-up. Furthermore, prospective randomized studies are necessary to assess whether Mosh surgery may be superior to standard treatments and whether ART therapy may be superior to surgery alone in patients with PNI.

Finally, a better histological definition and the identification of PNI related biomarkers could improve knowledge on the pathogenetic mechanisms underlying PNI, contribute to a more precise prognostic stratification and reveal potential targets for the development of future therapies.

## Key References

- Stratigos AJ, Garbe C, Dessinioti C, Lebbe C, van Akkooi A, Bataille V, et al. European consensus-based interdisciplinary guideline for invasive cutaneous squamous cell carcinoma. Part 1: Diagnostics and prevention-Update 2023. Eur J Cancer. 2023;193:113251.
  - This reference is of particular importance because it shows the current treatment indications in patients with cSCC.
- Chung E, Hoang S, McEvoy AM, Rosman IS, Hurst EA, Council ML. Histopathologic upgrading of cutaneous squamous cell carcinomas during Mohs micrographic surgery: A retrospective cohort study. J Am Acad Dermatol. 2021;85:923–30.
  - This reference is of importance because because it shows that surgical treatment with the Mosh technique favors a more accurate diagnosis.
- Zhang J, Wang Y, Wijaya WA, Liang Z, Chen J. Efficacy and prognostic factors of adjuvant radiotherapy for cutaneous squamous cell carcinoma: A systematic review and meta-analysis. J Eur Acad Dermatology Venereol. 2021;35:1777–87.
  - This reference is of particular importance because collects all the evidence on the effectiveness post treatment radiotherapy, shown the complexity of reaching an unambiguos consensus.

## Data Availability

No datasets were generated or analysed during the current study.
